# Robotic Versus Conventional Nipple-Sparing Mastectomy With Immediate Breast Reconstruction

**DOI:** 10.3389/fonc.2021.637049

**Published:** 2021-03-04

**Authors:** Gilles Houvenaeghel, Julien Barrou, Camille Jauffret, Sandrine Rua, Laura Sabiani, Aurore Van Troy, Max Buttarelli, Guillaume Blache, Eric Lambaudie, Monique Cohen, Marie Bannier

**Affiliations:** ^1^ Department of Surgical Oncology, Paoli Calmettes Institute, Marseille, France; ^2^ CRCM, CNRS, INSERM, Aix Marseille Université, Marseille, France

**Keywords:** nipple sparing mastectomy, robotic surgery, breast reconstruction, predictive score, breast cancer

## Abstract

**Background:**

Several studies reported the feasibility and safety of robotic-NSM (R-NSM). The aim of our prospective study was to compare R-NSM and conventional-NSM (C-NSM).

**Methods:**

We analyzed patients who were operated on with and without robotic assistance (R-NSM or C-NSM) and who received immediate breast reconstruction (IBR) with implant or latissimus dorsi-flap (LDF). The main objective was complication rate and secondary aims were post-operative length of hospitalization (POLH), duration of surgery, and cost.

**Results:**

We analyzed 87 R-NSM and 142 C-NSM with implant-IBR in 50 and 135 patients, with LDF-IBR in 37 and 7 patients, respectively. Higher durations of surgery and costs were observed for R-NSM, without a difference in POLH and interval time to adjuvant therapy between R-NSM and C-NSM. In the multivariate analysis, R-NSM was not associated with a higher breast complication rate (OR=0.608) and significant factors were breast cup-size, LDF combined with implant-IBR, tobacco and inversed-T incision. Grade 2-3 breast complications rate were 13% for R-NSM and 17.3% for C-NSM, significantly higher for LDF combined with implant-IBR, areolar/radial incisions and BMI>=30. A predictive score was calculated (AUC=0.754). In logistic regression, patient’s satisfaction between C-NSM and R-NSM were not significantly different, with unfavorable results for BMI >=25 (OR=2.139), NSM for recurrence (OR=5.371) and primary breast cancer with radiotherapy (OR=4.533). A predictive score was calculated. In conclusion, our study confirms the comparable clinical outcome between C- NSM and R-NSM, in the price of longer surgery and higher cost for R-NSM. Predictive scores of breast complications and satisfaction were significantly associated with factors known in the pre-operative period.

## Introduction

Despite an increase in breast conservative surgery, a total mastectomy is still necessary in 12% to 30% of patients (1–3) in cases of extended ductal carcinoma in-situ (DCIS), invasive breast cancer (BC) with an extensive DCIS component, multifocal disease, large BC according to breast size without indication of neoadjuvant chemotherapy (NAC), prophylactic mastectomies, ipsilateral BC local recurrence (ILBCLR), non in-sano initial resection, and patient’s wishes. Immediate breast reconstruction (IBR) rate increased progressively in relation with patient’s wishes and better quality of life (4, 5).

Nipplesparing mastectomy (NSM) is associated with better aesthetic results and better quality of life than skin-sparing mastectomy (6, 7). Consequently, NSM is the procedure of choice when this technique is possible, mainly in relation to the tumor nipple-areolar-complex (NACx) distance on radiologic exams. Several studies reported a few cases of robotic-NSM (R-NSM) to evaluate feasibility, reproducibility, and safety (8–27). Recently, a technical robotic surgical consensual NSM procedure was reported (28). However, comparison between R-NSM and conventional- NSM (C-NSM) has been recently reported in only one retrospective study with a small sample size and for procedures realized by only one surgeon (29). Moreover, a recent US FDA safety communication (30), in February 2019 (US) does not validate this technique which required more data before new evaluation.

The aim of our prospective study was to compare R-NSM and C-NSM in terms of breast complication rate as a main objective, and hospital stay, duration of surgery, cost evaluation, and patient’s satisfaction as secondary objectives.

## Methods

### Patients

Robotic NSM and IBR were performed by two surgeons over 51 months (from the first procedure in November 2016 to March 2020). All patients were informed of robotic assistance surgery. Our institutional ethical committee approved robotic breast surgery procedures and data were collected in institutional breast database.

After the preliminary experience of R-NSM with 27 R-NSM (27), we determined a standardized technique with dissection with non-robotic scissors after sub cutaneous infiltration with adrenaline serum and then robotic dissection through a mono-trocar insert in axillary or lateral small incision (26, 27). This technique was reported as the consensus (28). In this prospective study, we analyzed patients operated on between March 2018 and March 2020, with and without robotic assistance (R-NSM and C-NSM) and IBR with implant breast or latissimus dorsiflap (LDF) with or without association with implant. The choice between R-NSM and C-NSM was determined by surgeons. The main objective was complication rate for R-NSM versus C-NSM and 240 patients were planned to achieve this comparison since March 2018, with a first hypothesis of 80 RNSM and 160 C-NSM. Secondary aims were: post-operative length of hospitalization (POLH), duration of surgical procedure, cost evaluation, and patient’s satisfaction according the two groups, R-NSM and C-NSM.

We determined the characteristics of patients (age, body mass index (BMI), tobacco use, diabetes, ASA status, breast cup-size), previous treatment for BC (sentinel lymph node biopsy, axillary lymph node dissection (ALND), neo-adjuvant chemotherapy, previous breast radiotherapy), indications of NSM (primary BC or local recurrence, reconstruction with robotic latissimus dorsi-flap (RLDF) and or breast implant). Interval times between surgery and adjuvant chemotherapy (AC) or post-mastectomy radiotherapy (PMRT) for patients without AC were recorded.

Surgical techniques with a type of Da Vinci system, number of trocars, skin incision, and duration of anesthesia and surgery were reported according to period of treatment and association of surgical procedures (mastectomy, breast implant, robotic-LDF (R-LDF), ALND, and contra-lateral breast surgery). Complication rate was determined by Clavien-Dindo grading (31) during a post-operative period of 30 days. Re-operation rate, type of complication, and number of POLH days were analyzed. During this period of study, we did not use an enhanced recovery program.

### Indications of NSM

NSM was proposed to patients for prophylactic mastectomy, for local recurrence when a second breast conservative treatment was not possible or not the patient’s choice and for primary BC with indication of total mastectomy. NSM was realized when the tumor-nipple distance was 1 centimeter or more on radiologic exams. A retro nipple-areolar complex biopsy was systematically performed with definitive pathologic exam without per-operative analysis. Incisions were determined by surgeons according to breast characteristics and usual practice of surgeons. Inversed-T incisions with C-NSM were used for high breast volume with ptotic breast (breast cup-size >C and nipple areolar complex under infra mammary fold). A pre-operative flap thickness was assessed by surgeons during a clinical exam and a digital mammogram.

### C-NSM Procedure

Depending on the surgeon’s habits, there was either infiltration with adrenalin serum, or just serum, or no infiltration at all. For superficial dissection, two techniques were used: scissors, or monopolar coagulation with electric usual coagulation or peak plasma blade. The exposition was done using retractors.

R-NSM procedure was reported previously (26, 27, 32). In summary, after superficial adrenal infiltration, dissection between skin and breast gland was conducted with scissors. Then, through a Gel point mono-trocar device disposed in axillar or external incision, two robotic trocars were inserted for the robotic camera (inferior part of Gel point) and one robotic instrument (superior part of Gel point). Another trocar was inserted at the inferior external part of breast (at the infra-mammary fold level). Complementary superficial dissection, periphery, and deep dissections were realized with robotic instruments (scissors with monopolar coagulation), using a low insufflation pressure (7mm Hg). When the implant was disposed under the pectoral muscle, the pocket was also performed with robotic instruments. Cost evaluation has been analyzed for all patients and for the following sub-populations: R-NSM and C-NSM, R-NSM with implant-IBR, and C-NSM with implant-IBR. Cost evaluation, expressed in Euros, was performed with cost of duration of anesthesia (length of operative room occupation), length of hospitalization (day number), cost of robotic instrumentation and other devices used, and cost of breast implant. We did not include purchase and maintenance costs of Da Vinci systems which are in relation with number of procedures per-year for breast surgery and others indications of robotic procedures for urologic, gynecologic and digestive tumors with a total of 862 procedures during the study period (98 patients for breast robotic surgery and 764 patients for others indications). In France, all fees for breast reconstruction are reimbursed by national insurance and cost of robotic procedure was supported by the institution. Patients did not have to pay out-of-pocket. Patient satisfaction was assessed by asking, orally by surgeons, at the consultation 6 to 12 months after surgery if satisfaction was very good, good, medium, fair, or unsatisfied.

### Statistics

Main characteristics were reported with median, mean, and confident interval 95% (CI 95) for quantitative criteria. Comparisons were performed using Chi2, t-test, and binary logistic regression adjusted to significant univariate variables, with SPSS 16.0 (SPSS Inc., Chicago, Illinois). Predictive scores were calculated using Odds Ratios (OR) determined by logistic regression and evaluated by calculation of area under the ROC curves (AUC).

## Results

**Figure 1:**
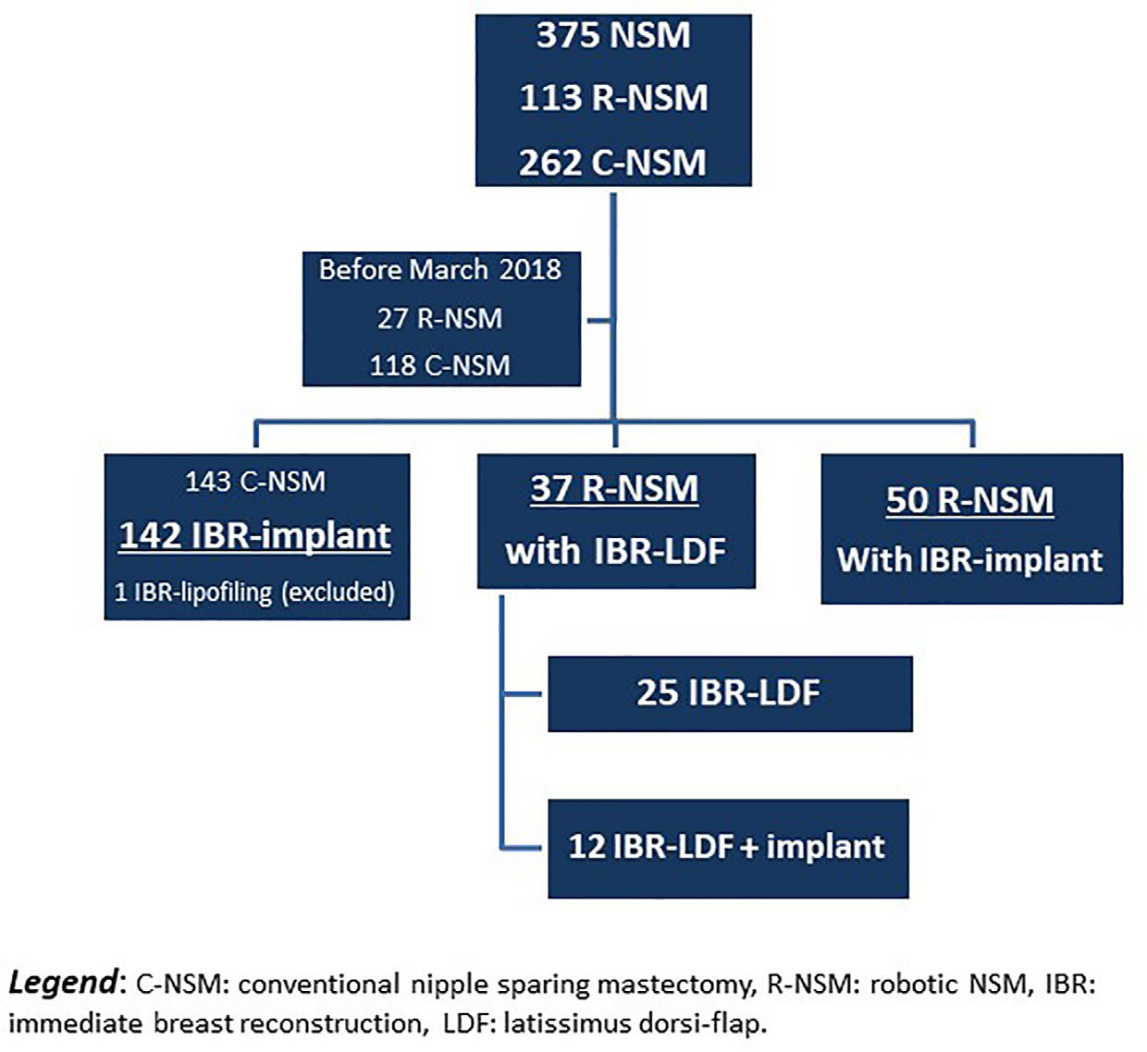
Flow chart of patients.

Out of 375 NSM performed since January 2016, 145 NSM were realized before March 2018, with 27 R-NSM reported in the preliminary experience of R-NSM. The present study analyzed 229 patients operated on from March 2018 to March 2020 (breast robotic surgery stopped on 2020-03-15 due to COVID-19 pandemic) with the exclusion of 1 patient with C-NSM and exclusive IBR-lipofilling (**Figure 1**). R-NSM were performed by two surgeons (82 and 5, respectively) and C-NSM (n=142) were performed by 8 surgeons (6 to 53 C-NSM). Characteristics of patients are reported in **Table 1** and **Supplemental Data File 1** according to technique used for NSM, R-NSM or C-NSM, and type of IBR (implant or expander, latissimus dorsi-flap). R-NSM with R-LDF-IBR was performed in 37 cases, with associated implant breast in 12 cases. All others R-NSM-IBR were realized with definitive breast implant (n=50) in 7 cases with pre-pectoral implant (3 prophylactic NSM and 4 for primary BC).

**Table 1:** Characteristics of patients.

		C-NSM		R-NSM		Chi2
		Nb	%	Nb	%	p
Number		142	62.0	87	38.0	
BMI	<= 24.9	119	83.8	73	83.9	0.825
	25-29.9	17	12.0	9	10.3	
	>= 30	6	4.2	5	5.7	
Cup size	A-B	92	64.8	49	56.3	0.393
	C	34	23.9	24	27.6	
	> C	16	11.3	14	16.1	
Tobacco	No	118	83.1	61	70.1	0.017
	Yes	24	16.9	26	29.9	
Indication	Primitive	75	52.8	70	80.5	<0.0001
	Local recurrence	7	4.9	10	11.5	
	Prophylactic	60	42.3	7	8.0	
IBR-Type	Implant	128	90.1	50	57.5	<0.0001
	Expander	7	4.9	0	0	
	LDF	6	4.2	25	28.7	
	LDF + implant	1	0.7	12	13.7	
Breast weight	<= 300	92	64.8	31	35.6	<0.0001
	> 300	50	35.2	56	64.4	
Complication breast	No	103	72.5	68	78.2	0.214
	Yes	39	27.5	19	21.8	
Complication dorsal	Yes	4	57.1	7	18.9	0.263
	No	3	42.9	30	81.1	
Previous radiotherapy	No	129	90.8	67	77.0	0.004
	Yes	13	9.2	20	23.0	
Radiotherapy	No	123	86.6	41	47.1	<0.0001
	PMRT	12	8.5	27	31.0	
	Previous RTH	6	4.2	10	11.5	
	NAC+NAR	1	0.7	9	10.3	
NAC	No	130	91.5	62	71.3	<0.0001
	Yes	12	8.5	25	28.7	

C-NSM, conventional nipple sparing mastectomy; R-NSM, robotic NSM; BMI, body mass index, DCIS, ductal carcinoma in situ; IBR, immediate breast reconstruction; LDF, latissimus dorsi-flap; POLH, post-operative length of hospitalization; PMRT, post-mastectomy radiotherapy; RTH, radiotherapy; NAC, neo-adjuvant chemotherapy; NAR, neo-adjuvant radiotherapy; SLNB, sentinel lymph node biopsy; ALND, axillary lymph node dissection.

Several significant differences were reported between two groups with higher risk factor rates in RNSM group for tobacco use, histology, primary BC and local recurrence, breast weight, and oncologic treatments (**Table 1**, **Supplemental Data Files 2**). Breast cancer treatment: In the R-NSM group, higher rates of adjuvant chemotherapy, neo-adjuvant chemotherapy, and endocrine therapy were observed in comparison with the C-NSM group. More PMRT, previous radiotherapy, and neo-adjuvant chemotherapy with neo-adjuvant radiotherapy were also reported (**Table 1**, **Supplemental Data Files 1**).

Durations of surgery included all procedures and several installations from skin incision to the end of skin suture for R-NSM with R-LDF-IBR. Mean duration of surgery for all patients was 174 minutes. Median and mean durations are reported in **Supplemental Data Files 2**, for all patients and according to surgical procedures with higher values for robotic procedures. Duration of surgery >180mn was significantly associated in univariate analysis with R-NSM (p<0.0001), type of incision (p=0.003), implant-IBR or LDF-IBR or LDF-IBR with implant (p<0.001), age (p<0.0001), axillary surgery type (p<0.0001), breast cup-size (p=0.002), previous radiotherapy (p<0.0001), neo-adjuvant chemotherapy (p<0.0001), ASA status (p=0.001), year of surgery (p=0.005), indication for prophylactic surgery or local recurrence or primary BC (p=0.021), and surgeons (p<0.0001). In binary logistic regression, duration of surgery >180mn was significantly associated with R-NSM (OR: 101, CI95% 6.59-1548).

### Outcome

Median POLH was 2 days: 3 days for R-NSM and 2 days for C-NSM without significant difference between C-NSM-implant and R-NSM-implant, and between C-NSM-LDF and R-NSM-LDF (**Supplemental Data Files 2**). *Overall breast complication* crude rate was 25.3%: 21.8% (CI95% 13.2-30.5) for R-NSM and 27.5% (CI95% 20.1-34.8) for C-NSM (p=0.214). For patients with R-NSM combined with LDF-IBR, overall complication rate was 23.3%, 19.0% for breast complications, and 70.0% for dorsal complications (85.7% Grade 1 dorsal complications: dorsal seroma) in comparison with 13.8% breast complications for R-NSM with implant-IBR. Overall breast complications rates according to type of incisions were significantly different with a higher complication rate for inversed-T incision and areolar/radial incisions (**Table 2**). Inversed-T incisions were significantly associated with high breast volume: breast cup-size (p=0.003), breast weight (p=0.001) and BMI (p=0.017). In regression analysis adjusted on significant factors in univariate analysis, R-NSM was not associated with a higher breast complication rate in comparison with C-NSM (OR: 0.608, CI95% 0.225-1.64, p=0.326). Significant factors of breast complications were breast cup-size, LDF-IBR with implant, tobacco, and inversed-T incision (**Table 3**).

**Table 2:** Breast complications and Grade 2-3 breast complications.

		Breast complication			Chi2	Grade 2-3 breast complication			
		No	Yes	%	p	No	Yes	%	p
Number		171	58	25.3		198	31	13.5	
BMI	<= 24.9	147	45	23.4	0.210	171	21	10.9	**0.018**
	25-29.9	18	8	30.8		20	6	23.1	
	>= 30	6	5	45.5		7	4	36.4	
Cup size	A-B	115	26	18.4	**0.010**	128	13	9.2	**0.009**
	C	37	21	36.2		49	9	15.5	
	> C	19	11	36.7		21	9	30.0	
Tobacco	No	140	39	21.8	**0.018**	157	22	12.3	0.206
	Yes	31	19	38.0		41	9	18.0	
IBR-Type	Implant-Expander	139	46	24.9	**0.030**	162	23	12.4	**0.001**
	LDF	26	5	16.1		29	2	6.5	
	LDF + implant	6	7	53.8		7	6	46.2	
Implant size	<= 300	93	22	19.1	**0.002**	107	8	7.0	**<0.0001**
	> 300	44	29	39.7		53	20	27.4	
Breast weight	<= 300	99	24	19.5	**0.021**	114	9	7.3	**0.003**
	> 300	72	34	32.1		84	22	20.8	
Previous radiotherapy	No	148	48	24.5	0.304	173	23	11.7	**0.054**
	Yes	23	10	30.3		25	8	24.2	
Surgeons					0.197				**0.041**
Incision	Peripheric	108	29	21.2	**0.002**	124	13	9.5	**0.020**
	Areolar - radial	47	18	27.7		52	13	20.0	
	Previous incision	13	3	18.8		15	1	6.2	
	Inversed T	3	8	72.7		7	4	36.4	
NSM Type	C-NSM	103	39	27.5	0.214	121	21	17.3	0.309
	R-NSM	68	19	21.8		77	10	13.0	
NSM and IBR types	C-NSM-implant	97	38	28.1	0.296	115	20	14.8	0.316
	C-NSM-LDF	6	1	14.3		6	1	14.3	
	R-NSM-implant	26	11	29.7		30	7	18.9	
	R-NSM-LDF	42	8	16.0		47	3	6.0	

C-NSM, conventional nipple sparing mastectomy; R-NSM, robotic NSM; BMI, body mass index; IBR, immediate breast reconstruction; LDF, latissimus dorsi-flap; POLH, post-operative length of hospitalization. Bold values statistical significant results.

**Table 3:** Breast complications and Grade 2-3 breast complications: Binary logistic regression.

Complications		p	OR	CI 95%	
				Inferior	Superior
**Breast complications**					
Cup size	A-B		1		
	C	0.003	3.158	1.462	6.82
	> C	0.322	1.714	0.59	4.975
Tobacco	Yes vs no	0.001	3.86	1.742	8.554
IBR-type	Implant		1		
	LDF	0.42	0.616	0.19	1.999
	LDF+implant	0.033	4.502	1.129	17.95
NSM type	R-NSM vs C-NSM	0.326	0.608	0.225	1.64
Incisions	Peripheric		1		
	Areolar / radial	0.74	1.161	0.481	2.806
	Previous BCS incision	0.514	0.602	0.131	2.765
	Inversed T	0.004	10.51	2.131	51.86
**Grade 2-3 breast complications**					
Cup size	A-B		1		
	C	0.666	0.782	0.256	2.391
	> C	0.322	1.846	0.549	6.206
IBR-type	Implant		1		
	LDF	0.158	0.269	0.044	1.661
	LDF+implant	0.045	5.672	1.041	30.92
NSM type	R-NSM vs C-NSM	0.913	1.09	0.229	5.188
Incisions	Peripheric		1		
	Areolar / radial	0.026	4.574	1.197	17.47
	Previous BCS incision	0.733	0.66	0.06	7.205
	Inversed T	0.063	5.647	0.908	35.11
BMI	<= 24.9		1		
	25-29.99	0.118	2.698	0.778	9.36
	>= 30	0.026	7.429	1.278	43.17
Previous RTH	Yes vs no	0.094	2.865	0.836	9.821
Surgeons		0.665	0.957	0.786	1.166

We chose for adjustment breast cup-size which is known before surgery. A strong correlation between breast cup-size and BMI, breast weight was observed. C-NSM, conventional nipple sparing mastectomy; R-NSM, robotic NSM; BMI, body mass index; IBR, immediate breast reconstruction; LDF, latissimus dorsi-flap; RTH, radiotherapy.

*Grade 2-3 breast complications* rate was 13.5%, 13% for R-NSM (CI95% 4.8-18.2) and 17.3% for CNSM (CI95% 8.95-20.63) (**Table 2**). Grade 2-3 breast complications rates according to type of incisions were significantly different with higher complication rates for inversed-T incision and areolar/radial incisions (**Table 2**). Re-operation rates were not significantly different between two groups of NSM (p=0.583).

**Figure 2:**
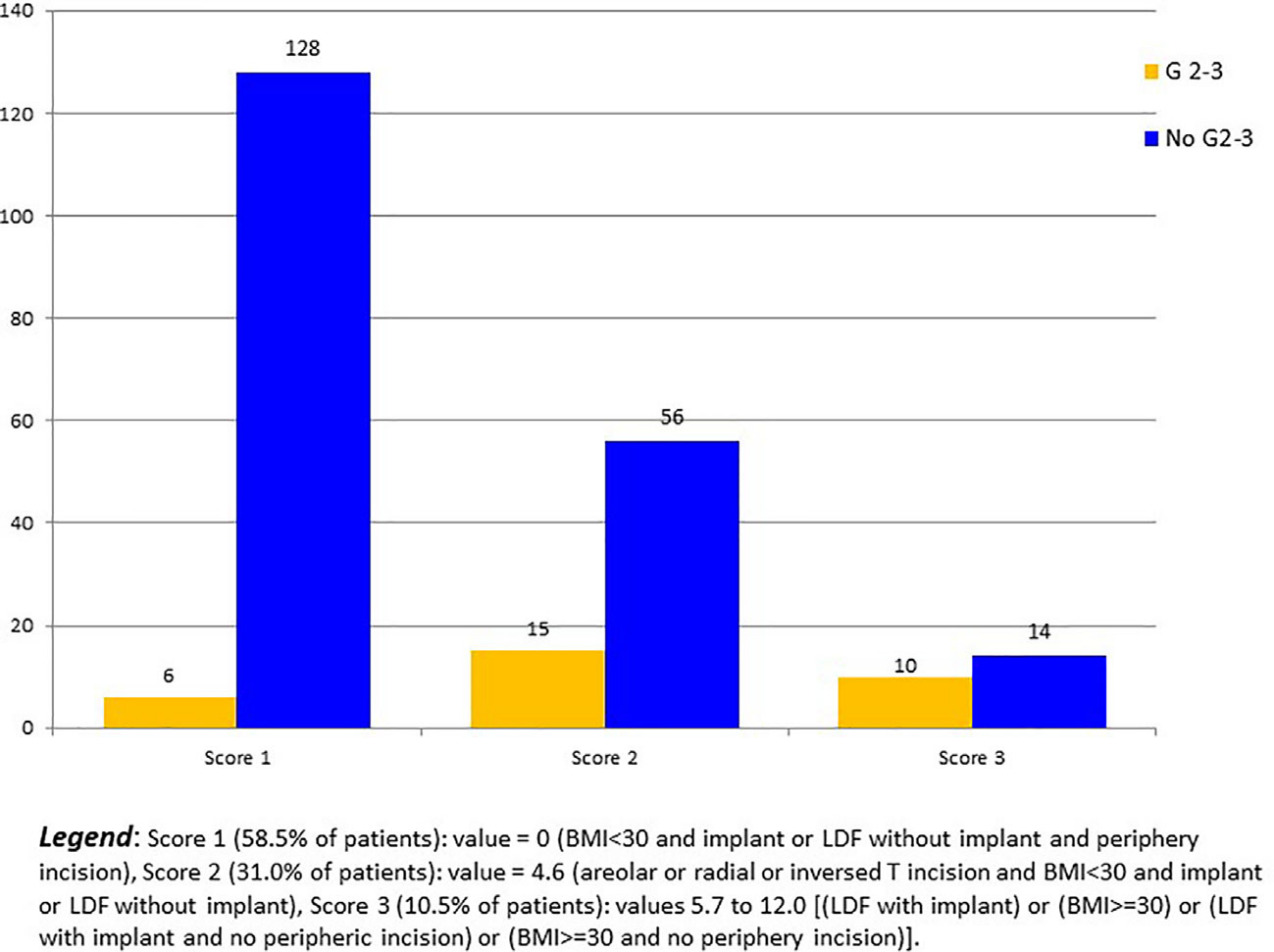
Grade 2-3 breast complication numbers according to 3 scores values.

In regression analysis, R-NSM was not associated with a higher Grade 2-3 breast complication rate in comparison with C-NSM (OR: 1.09, CI95% 0.229-5.188, p=0.913). Significant factors of Grade 2-3 breast complications were LDF-IBR with implant, areolar/radial incisions, and BMI >=30 (**Table 3**). Predictive score of breast complication Grade 2-3 was calculated (BMI >=30: 7.4 versus 0, LDF with implant: 5.7 versus 0, areolar or radial or inversed T incisions: 4.6 versus 0) with breast complications Grade 2-3 rates of 4.5% (6/134), 21.1% (15/71), 50.0% (6/12), 28.6% (2/7), 0% (0/1) and 50.0% (2/4) for score values 0, 4.6, 5.7, 7.4, 10.3 and 12.0, respectively (p<0.0001) (**Figure 2**). AUC of ROC curve was 0.754 (CI95% 0.660-0.847).

Implant loss rate was 7.8%: 10.2% for R-NSM (CI95% 2.5-17.9) (2.1% for R-NSM-implant and 41.7% for R-NSM-LDF-implant: 5/12) and 6.7% for C-NSM (CI95% 2.2-11.1) (p=0.293). High BMI (4 patients >24.9), high breast cup-size (8>=C), and high mastectomy weight (9>470gr) were observed among the twelve patients with R-NSM-LDF-implant.

Types of complications according to Grade of complication are reported in **Supplemental Data Files 3**. The more frequent complications were NACx or skin-flap suffering or necrosis (56.9%) and hematomas. Types of complications were not significantly different between C-NSM and R-NSM (p=0.770).

### Interval Time Between Surgery to Adjuvant Therapy

There was no difference between C-NSM and RNSM for interval time <= or >60 days (p=0.530) and median interval time (**Supplemental Data Files 2**). For adjuvant chemotherapy and PMRT, median interval times were 48 days (mean 51, CI95% 41.9-60.2) and 60 days (mean 67.8, CI95% 53.5-82.1) respectively (p=0.042), 23.5% >60 days for chemotherapy and 46.7% for PMRT.

### Cost Evaluation

Significantly higher cost was observed for R-NSM versus C-NSM. Mean cost was higher (+34.7%: 1749 Euros) for R-NSM-implant versus C-NSM-implant and higher (+30%: 2357 Euros) for RNSM-LDF versus C-NSM-LDF (**Supplemental Data Files 2**).

### Patient’s Satisfaction

**Figure 3:**
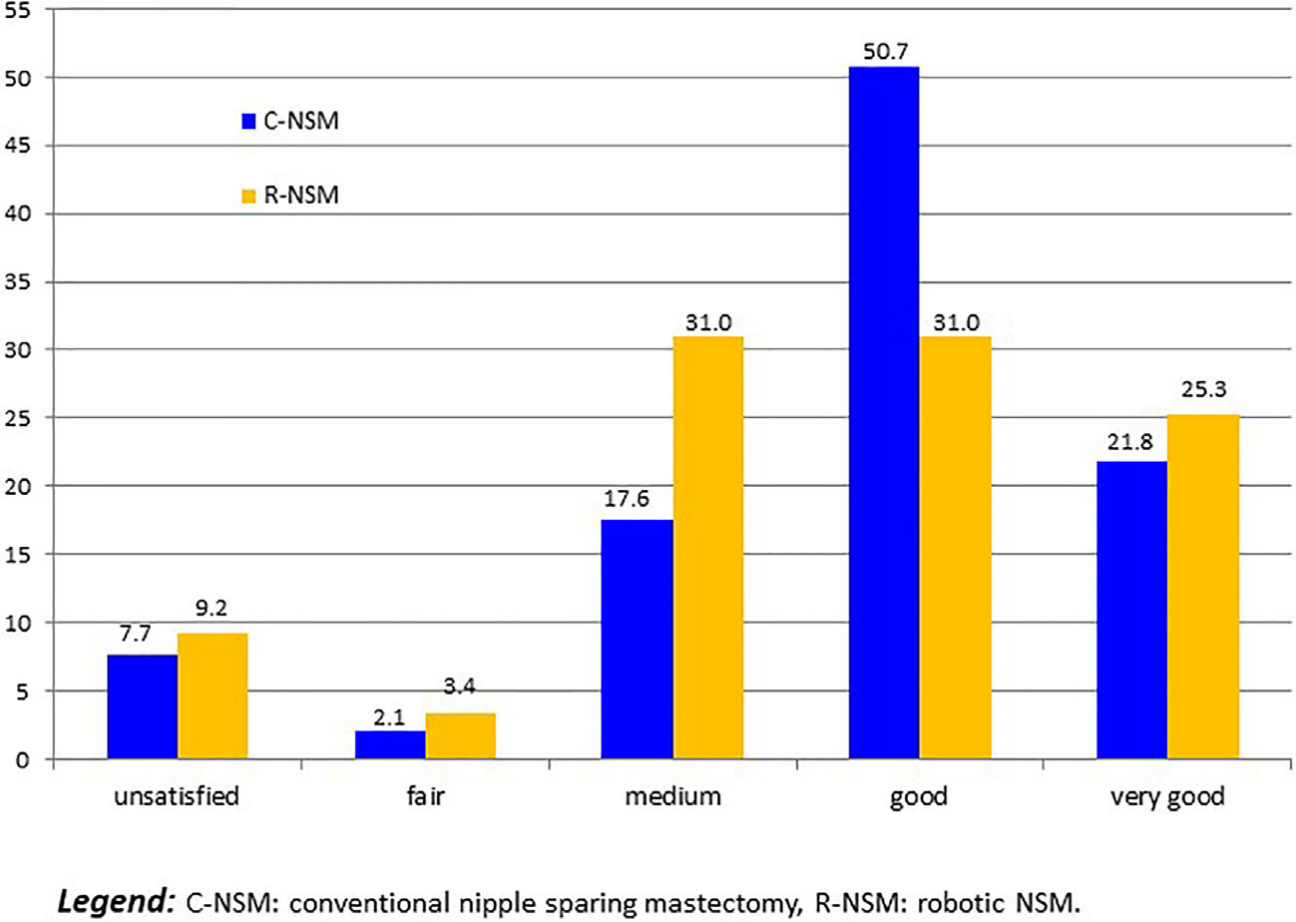
Patient satisfaction according to C-NSM and R-NSM (number of patients).

**Figure 4:**
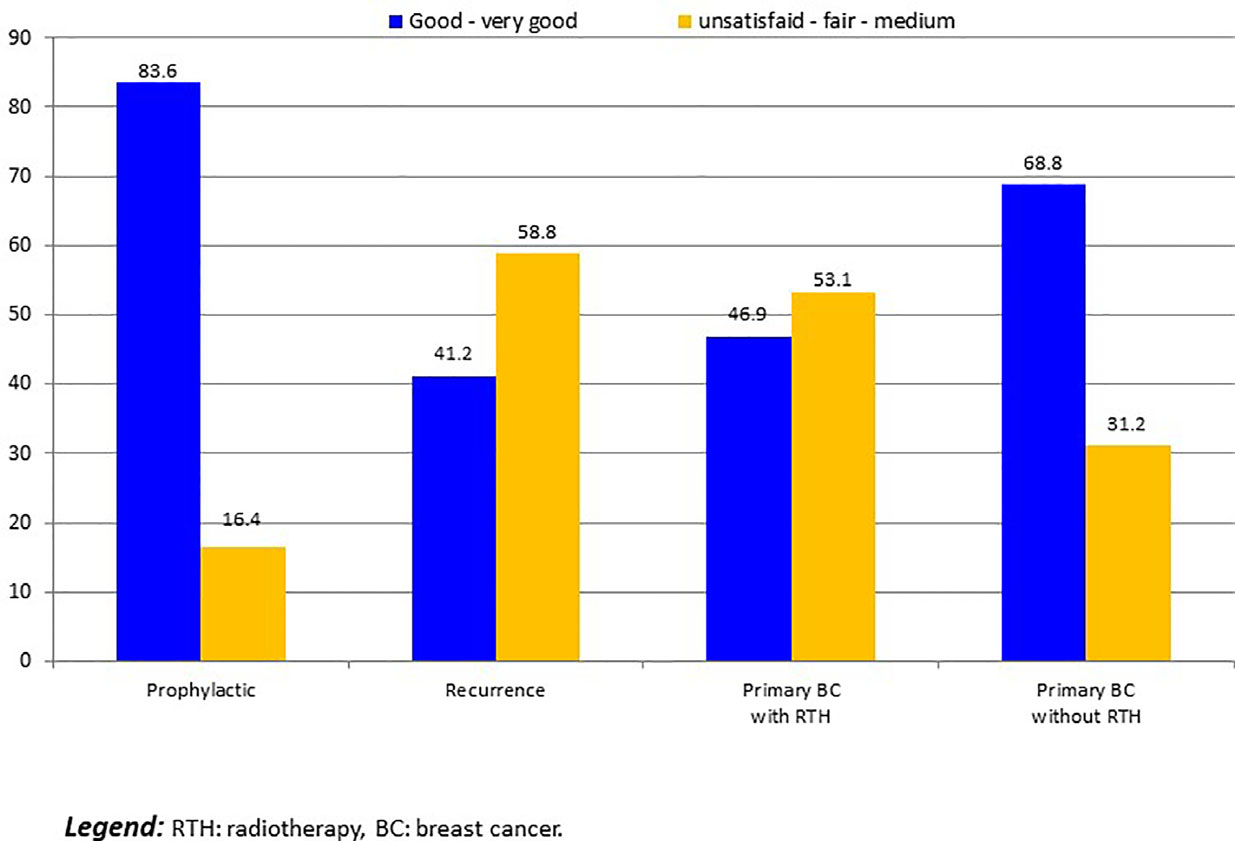
Patient satisfaction according to indication of NSM and radiotherapy (number of patients).

For 14 patients with implant loss, we considered that patients were unsatisfied. Five others patients required delayed explantation and were classified as unsatisfied: 2 R-NSM-implant for secondary complication with interval between IBR and explantation of more than 1 year, and 3 C-NSM-implant for local recurrence in 2 cases and patient’s choice in 1 case. Satisfaction was: 19 unsatisfied (8.3%:19/229), 6 fair (2.6%), 52 medium (22.7%), 99 good (43.2%) and 53 very good (23.1%). Satisfactions according to groups C-NSM and R-NSM were better for C-NSM (p=0.042) (**Figure 3**). In univariate analysis, satisfaction with 2 groups (>= or < good) were associated with BMI (p=0.012), tobacco (p=0.013), indication and radiotherapy (p=0.001) (**Figure 4**) and C-NSM or R-NSM (p=0.009). In binary logistic regression, adjusted on tobacco, indication and radiotherapy, BMI, C-NSM, and R-NSM were not significantly different (OR: 1.151, CI95% 0.591-2.241, p=0.679) with unfavorable results for BMI >=25 (OR: 2.139, CI95% 0.996-4.595, p=0.051), NSM for recurrence (OR: 5.371, CI95% 1.560-18.49, p=0.008) and primary BC with radiotherapy (OR: 4.533, CI95% 1.728-11.89, p=0.002) (non-significant: tobacco and primary BC without radiotherapy in comparison with prophylactic NSM). Predictive score of satisfaction good or very good (versus others) was calculated (BMI >=25: 2 versus 0, recurrence: 5.0 and primary BC with radiotherapy: 4.5 (versus 0 for prophylactic and primary BC without radiotherapy)) with unsatisfied or bad or medium results in 24.8%, 27.3%, 46.2%, 41.7%, 80.0% and 100% for score values of 0 (141 patients: 61.6%), 2.0 (22 patients: 9.6%), 4.5 (39 patients: 17.0%), 5.0 (12 patients: 5.2%), 6.5 (10 patients: 5.2%) and 7.0 (5 patients: 2.2%), respectively (p<0.0001). AUC of ROC curve was 0.651 (CI95% 0.572-0.730).

## Discussion

Despite several significant higher rates of risk factors for complications in the R-NSM group in comparison with C-NSM group, we reported no significant difference for breast complications and Grade 2-3 breast complications in univariate and multivariate analysis between the two groups. There was also no significant difference for POLH and interval time before adjuvant treatments. However, significant higher durations of surgery and costs were observed for the R-NSM group. Since the first publications, R-NSM were reported in several studies to determine the feasibility and technique, including 1 to 94 procedures (8–27). Despite these reports, FDA safety communication in February 2019 underlined caution when using robotically-assisted surgical devices for mastectomy (30).

In a recent study by Lai et al. (29), a comparison between R-NSM and C-NSM with implant IBR was reported to determine complication rates, duration of surgery, and costs. During a period of 99 months, authors reported 54 R-NSM and 62 C-NSM. In our study, we reported higher numbers of patients in each group, 87 R-NSM and 142 C-NSM, during a shorter period of 25 months. A lower RNSM rate of 38.0% in our study was reported in comparison with 46.5% in the Lai et al. study.

Complication rates were not significantly different between two groups (29) as we observed. Complication rates were 41% and 46.8% in the Lai et al. study (29), 21.8% and 27.5% in our study, for RNSM and C-NSM groups, respectively. The rate of reoperation was 4.3% in the Toesca et al. study among 73 women who underwent 94 R-NSM (10), lesser than our results of 9.2% in each groups. Breast complications Grade 2-3 could be predicted using our score with a good accuracy (AUC: 0.754), with low rates of Grade 2-3 breast complications for patients with BMI<30 and periphery incision and implant-IBR or LDF-IBR without implant (4.48%) and for patients with BMI <30 and implant-IBR or LDF-IBR without implant and no periphery incision (21.1%) in comparison with a rate of 41.7% for other patients. However, this score needs validation in another independent study.

Hospital stays were higher in the Lai et al. study (29) for all patients and the two groups in comparison with our practice: 6 days versus 2 days in our study. This might be correlated with the Enhanced Recovery after Surgery program, set up in our institute in 2017, initially for gynecologic, digestive, and urologic surgery. Another important difference was mean mastectomy weights which were 293gm and 386gm for R-NSM, 321 and 280gm for C-NSM, in the Lai et al. study (29) and in our study, respectively.

As we reported, higher durations of surgery were observed (29) for R-NSM with a difference of 27mn between the two groups, corresponding to increase of 12% of time for R-NSM versus C-NSM. We reported higher differences: an increase of 41.5% for R-NSM-implant versus C-NSM-implant and 28% for R-NSM-LDF versus C-NSM-LDF. However, the mean durations of surgeries were comparable: 224 and 184mn for R-NSM-implant, 197 and 130mn for C-NSM in the Lai et al. study (29) versus our study respectively.

Cost evaluation in the Lai et al. study (29) differed between two groups with higher total costs for R-NSM versus C-NSM: 90.7% more expensive for R-NSM in comparison with C-NSM. In our study, R-NSM was also more expensive but with a difference of 34.7% for R-NSM-implant and 30% for R-NSM-LDF. However, the method of costs evaluations differed between the two studies. The overall satisfaction rate was higher in the R-NSM group versus C-NSM group in study of Lai et al. (29) mainly attributed with periphery breast scar location for R-NSM. In our study, there was no significant difference between C-NSM and R-NSM in multivariate analysis and unfavorable satisfaction results could be predicted using our score with intermediate accuracy (AUC=0.651). However, good and very good results were reported in 74.8% of patients with score value <=2 (BMI <25 without other pejorative factor) which represented 71.2% of patients and in 54.9% of patients with score value 4.5 or 5.0 (primary BC with radiotherapy or recurrence and BMI <25) which represented 22.2% of patients.

We determined several situations with factors known in the pre-operative period associated with high rates of breast complications (tobacco use, IBR with LDF and implant, cup-size >= C, BMI >=30 and incisions with inversed-T or areolar/radial) and a predictive pre-operative score of Grade 2-3 complications with a contributive accuracy (AUC=0.754). In these situations, information for patients should be completed before the patient’s choice for IBR or no IBR. Despite a greater number of patients in comparison with the first reported study comparing C-NSM and R-NSM (29), our study is limited in its small sample size and satisfaction evaluation performed at different intervals between surgery and last follow-up, without use of a validated questionnaire and without aesthetic assessment using photography before and after breast reconstruction.

Selection bias between two groups, C-NSM versus R-NSM, was compassed by multivariate analysis. Oncological results cannot be analyzed due to the very short follow-up period. In conclusion, our study confirms comparable clinical outcomes between conventional NSM and robotic NSM, with a greater number of patients than the previous study, at the cost of longer surgery and higher cost for robotic surgery. Interestingly, predictive scores of breast complications and satisfaction were significantly associated with factors known in the pre-operative period.

## Data Availability Statement

The original contributions presented in the study are included in the article/**Supplementary Material**. Further inquiries can be directed to the corresponding author.

## Ethics Statement

The studies involving human participants were reviewed and approved by Paoli Calmettes Institute review board. The patients/participants provided their written informed consent to participate in this study.

## Author Contributions

Authors who made substantial contributions to conception and design, and/or acquisition of data, and/or analysis and interpretation of data: GH, JB, CJ, MC, and MBa. Authors who participated in drafting the article or revising it critically for important intellectual content: GH, JB, MC, and MBa. Authors who gave final approval of the version to be published: GH, JB, CJ, SR, LS, AV, MBu, GB, EL, MC, and MBa. All authors contributed to the article and approved the submitted version.

## Conflict of Interest

The authors declare that the research was conducted in the absence of any commercial or financial relationships that could be construed as a potential conflict of interest.
