# Supplementary material for: Robotic Versus Conventional Nipple-Sparing Mastectomy With Immediate Breast Reconstruction
Source: Front Oncol. 2021 Mar 4;11:637049. doi: 10.3389/fonc.2021.637049 (PMC7971115; doi:10.3389/fonc.2021.637049)
Supplement: Supplementary file 1 [file Table_1.docx]

**Supplemental data files 1: Characteristics of patients (continue)**

|  |  | C-NSM |  | R-NSM |  | Chi2 |
| --- | --- | --- | --- | --- | --- | --- |
|  |  | Nb | % | Nb | % | p |
| ASA status | 1 | 67 | 47.2 | 33 | 37.9 | 0.305 |
|  | 2 | 72 | 50.7 | 53 | 60.9 |  |
|  | 3 | 3 | 2.1 | 1 | 1.1 |  |
| Histology | Lobular | 15 | 10.8 | 15 | 17.2 |  |
|  | Others | 0 | 0 | 1 | 1.1 |  |
|  | benign | 54 | 38.8 | 7 | 8.0 |  |
| Complication Grade | 0 | 103 | 72.5 | 68 | 78.2 | 0.693 |
| breast | 1 | 18 | 12.7 | 9 | 10.3 |  |
|  | 2 | 7 | 4.9 | 2 | 2.3 |  |
|  | 3 | 14 | 9.9 | 8 | 9.2 |  |
| Re-operation | No | 129 | 90.8 | 79 | 90.8 | 0.583 |
|  | Yes | 13 | 9.2 | 8 | 9.2 |  |
| Implant size | <= 300 | 85 | 68.0 | 30 | 47.6 | 0.006 |
|  | > 300 | 40 | 32.0 | 33 | 52.4 |  |
| Complication Grade | 0 | 103 | 72.5 | 68 | 78.2 | 0.693 |
| breast | 1 | 18 | 12.7 | 9 | 10.3 |  |
|  | 2 | 7 | 4.9 | 2 | 2.3 |  |
|  | 3 | 14 | 9.9 | 8 | 9.2 |  |
| Re-operation | No | 129 | 90.8 | 79 | 90.8 | 0.583 |
|  | Yes | 13 | 9.2 | 8 | 9.2 |  |
| Implant loss | Yes | 8 | 6.7 | 6 | 10.2 | 0.293 |
|  | No | 112 | 93.3 | 53 | 89.8 |  |
| PLOH | <= 3 | 117 | 82.4 | 60 | 69.0 | 0.015 |
|  | > 3 | 25 | 17.6 | 27 | 31.0 |  |
| Duration surgery | <= 180 mn | 123 | 86.6 | 31 | 35.6 | <0.0001 |
|  | > 180 mn | 19 | 13.4 | 56 | 64.4 |  |
| Chemotherapy | No | 125 | 88.0 | 68 | 78.2 | 0.037 |
|  | Yes | 17 | 12.0 | 19 | 21.8 |  |
| Endocrine therapy | No | 89 | 62.7 | 33 | 37.9 | <0.0001 |
|  | Yes | 53 | 37.3 | 54 | 62.1 |  |
| Axillary surgery | No | 78 | 54.9 | 33 | 37.9 | <0.0001 |
|  | SLNB | 59 | 41.5 | 37 | 42.5 |  |
|  | ALND | 5 | 3.5 | 17 | 19.5 |  |
| Interval time therapy | <= 60 days | 13 | 72.2 | 22 | 68.8 | 0.530 |
|  | > 60 days | 5 | 27.8 | 10 | 31.2 |  |
| year of surgery | 2018 | 52 | 36.6 | 48 | 55.2 | 0.005 |
|  | 2019 | 71 | 50.0 | 36 | 41.4 |  |
|  | 2020 | 19 | 13.4 | 3 | 3.4 |  |
| surgeons |  |  |  |  |  | <0.0001 |
| incision | peripheric | 54 | 38.0 | 83 | 95.4 | <0.0001 |
|  | areolar - radial | 64 | 45.1 | 1 | 1.1 |  |
|  | previous incision | 13 | 9.2 | 3 | 3.4 |  |
|  | inversed T | 11 | 7.7 | 0 | 0 |  |
