# Supplementary material for: Robotic Versus Conventional Nipple-Sparing Mastectomy With Immediate Breast Reconstruction
Source: Front Oncol. 2021 Mar 4;11:637049. doi: 10.3389/fonc.2021.637049 (PMC7971115; doi:10.3389/fonc.2021.637049)
Supplement: Supplementary file 2 [file Table_2.docx]

**Supplemental data files 2** : Median, Mean values according to C-NSM and R-NSM.

|  |  | Median | Mean | CI 95% | p |
| --- | --- | --- | --- | --- | --- |
| age | all | 48 | 49.6 | 48.1-51.2 |  |
|  | C-NSM | 46 | 47.8 | 45.9-49.7 | 0.002 |
|  | R-NSM | 51.5 | 52.7 | 50.1-55.4 |  |
|  | C-NSM-implant* | 46.5 | 47.6 | 45.6-49.5 | *0.008 |
|  | C-NSM-LDF° | 46.0 | 51.3 | 41.4-61.2 | °0.733 |
|  | R-NSM-implant* | 52.0 | 52.5 | 49.1-55.9 |  |
|  | R-NSM-LDF° | 51.0 | 53.0 | 48.8-57.3 |  |
| BMI | all | 21.1 | 22.2 | 21.8-22.7 |  |
|  | C-NSM | 21.0 | 21.8 | 21.2-22.4 | 0.016 |
|  | R-NSM | 22.4 | 22.9 | 22.2-23.7 |  |
|  | C-NSM-implant* | 20.9 | 21.6 | 21.0-22.2 | *0.430 |
|  | C-NSM-LDF° | 24.0 | 24.9 | 20.7-29.1 | °0.670 |
|  | R-NSM-implant* | 21.4 | 22.0 | 21.1-22.9 |  |
|  | R-NSM-LDF° | 23.5 | 24.2 | 23.0-25.5 |  |
| Duration | all | 149 | 174 | 164-185 |  |
| surgery | C-NSM | 130 | 135.8 | 128-144 | <0.0001 |
|  | R-NSM | 224 | 238.2 | 220-257 |  |
|  | C-NSM-implant* | 130 | 130.3 | 123-137 | *<0.0001 |
|  | C-NSM-LDF° | 244 | 242.4 | 204-281 | °0.012 |
|  | R-NSM-implant* | 173 | 183.8 | 168-200 |  |
|  | R-NSM-LDF° | 306 | 310.2 | 288-332 |  |
| Implant size | all | 280 | 288 | 276-301 |  |
|  | C-NSM | 280 | 278 | 263-293 | 0.036 |
|  | R-NSM | 320 | 309 | 284-333 |  |
|  | C-NSM-implant* | 280 | 278 | 263-293 | *0.072 |
|  | C-NSM-LDF | 320 | 320 |  |  |
|  | R-NSM-implant* | 320 | 307 | 278-337 |  |
|  | R-NSM-LDF | 325 | 314 | 277-352 |  |
| Breast weight | all | 287 | 320 | 295-344 |  |
|  | C-NSM | 236 | 280 | 250-309 | <0.0001 |
|  | R-NSM | 377 | 386 | 346-426 |  |
|  | C-NSM-implant* | 231 | 272 | 243-301 | *0.049 |
|  | C-NSM-LDF° | 337 | 427 | 190-664 | °0.699 |
|  | R-NSM-implant* | 344 | 332 | 282-381 |  |
|  | R-NSM-LDF° | 447 | 458 | 398-518 |  |
| POLH | all | 2 | 2.62 | 2.43-2.82 |  |
|  | C-NSM | 2 | 2.42 | 2.18-2.66 | 0.011 |
|  | R-NSM | 3 | 2.97 | 2.66-3.27 |  |
|  | C-NSM-implant* | 2 | 2.37 | 2.12-2.62 | *0.436 |
|  | C-NSM-LDF° | 3 | 3.43 | 2.53-4.33 | °0.342 |
|  | R-NSM-implant* | 2 | 2.22 | 1.84-2.51 |  |
|  | R-NSM-LDF° | 4 | 3.95 | 3.49-4.40 |  |
| Interval time | all | 51 | 55.9 | 48.2-63.5 |  |
| therapy | C-NSM | 51 | 56.6 | 44.0-69.1 | 0.889 |
|  | R-NSM | 51 | 55.4 | 45.2-65.7 |  |
|  | C-NSM-implant* | 55 | 58.9 | 45.3-72.6 | *0.315 |
|  | C-NSM-LDF° | 37.5 | 37.5 | 0-170 | °0.274 |
|  | R-NSM-implant* | 48 | 50.6 | 39.8-61.4 |  |
|  | R-NSM-LDF° | 60 | 66.7 | 40.9-92.4 |  |
| Cost | all | 5948 | 6345 | 5977-6713 |  |
|  | C-NSM | 4512 | 5180 | 4789-5571 | <0.0001 |
|  | R-NSM | 7929 | 8247 | 7719-8774 |  |
|  | C-NSM-implant* | 4499 | 5041 | 4648-5435 | *<0.0001 |
|  | C-NSM-LDF° | 7626 | 7858 | 6440-9276 | °0.004 |
|  | R-NSM-implant* | 6652 | 6790 | 6299-7281 |  |
|  | R-NSM-LDF° | 10071 | 10215 | 9580-10850 |  |

***Legend***: C-NSM: conventional nipple sparing mastectomy, R-NSM: robotic NSM, BMI : body mass index, LDF: latissimus dorsi-flap, POLH: post-operative length of hospitalization. Age: years, duration of surgery: minutes, implant size: centimeters^3^, breast weight: grams, POLH: days, interval time: days, cost: Euros.
