# Supplementary material for: Robotic Versus Conventional Nipple-Sparing Mastectomy With Immediate Breast Reconstruction
Source: Front Oncol. 2021 Mar 4;11:637049. doi: 10.3389/fonc.2021.637049 (PMC7971115; doi:10.3389/fonc.2021.637049)
Supplement: Supplementary file 3 [file Table_3.docx]

**Supplemental data files 3 :** Types and grades of complications.

|  | Types of complications | Grade 1 | Grade 2 | Grade 3 | Total Nb |
| --- | --- | --- | --- | --- | --- |
| C-NSM | hematoma | 0 | 0 | 7 | 7 |
|  | infection | 0 | 1 | 2 | 3 |
|  | NACx or Skin suffering | 15 | 4 | 5 | 24 |
|  | others | 3 | 2 | 0 | 5 |
| R-NSM | hematoma | 3 | 0 | 3 | 6 |
|  | infection | 0 | 0 | 2 | 2 |
|  | NACx or Skin suffering | 4 | 2 | 3 | 9 |
|  | others | 2 | 0 | 0 | 2 |
| Total | Nb | 27 | 9 | 22 | 58 |
|  | % | 46.6 | 15.5 | 37.9 |  |

***Legend***: C-NSM: conventional nipple sparing mastectomy, R-NSM: robotic NSM, Nb: number, NACx: nipple areolar complex.
